# An Update on the Tissue Renin Angiotensin System and Its Role in Physiology and Pathology

**DOI:** 10.3390/jcdd6020014

**Published:** 2019-03-29

**Authors:** Ali Nehme, Fouad A. Zouein, Zeinab Deris Zayeri, Kazem Zibara

**Affiliations:** 1 EA4173, Functional genomics of arterial hypertension, Univeristy Claude Bernard Lyon-1 (UCBL-1), 69008 Lyon, France; ali.hassan.nehme@gmail.com; 2 Department of Pharmacology and Toxicology, Heart Repair Division, Faculty of Medicine, American University of Beirut, Beirut 11-0236, Lebanon; fz15@aub.edu.lb; 3 Thalassemia & Hemoglobinopathy Research Center, Health Research Institute, Ahvaz Jundishapur University of Medical Sciences, Ahvaz, Iran; zeynabderisgenetice@gmail.com; 4 PRASE, Biology Department, Faculty of Sciences-I, Lebanese University, Beirut, Lebanon

**Keywords:** renin-angiotensin-aldosterone system, tissue, expression, physiology

## Abstract

In its classical view, the renin angiotensin system (RAS) was defined as an endocrine system involved in blood pressure regulation and body electrolyte balance. However, the emerging concept of tissue RAS, along with the discovery of new RAS components, increased the physiological and clinical relevance of the system. Indeed, RAS has been shown to be expressed in various tissues where alterations in its expression were shown to be involved in multiple diseases including atherosclerosis, cardiac hypertrophy, type 2 diabetes (T2D) and renal fibrosis. In this chapter, we describe the new components of RAS, their tissue-specific expression, and their alterations under pathological conditions, which will help achieve more tissue- and condition-specific treatments.

## 1. Introduction

In its classical view, RAS was defined as an endocrine system involved in blood pressure regulation and body electrolyte balance. However, RAS is now considered a “ubiquitous” system that is expressed locally in various tissues and exerts multiple paracrine/autocrine effects involved in tissue physiology and homeostasis [1]. Indeed, RAS plays key roles in cellular growth, proliferation, differentiation, migration, and apoptosis, in addition to extracellular matrix (ECM) remodeling and inflammation [2].

Alterations in RAS expression were shown to be involved in multiple diseases including atherosclerosis, cardiac hypertrophy, type 2 diabetes, and renal fibrosis [2,3,4]. On the other hand, RAS-blocking agents, such as angiotensin converting enzyme (ACE) inhibitors and AT1 receptor blockers (ARBs), have been shown to be effective in the management of hypertension-related cardiovascular diseases and end-organ damage [5]. Therefore, it is necessary to know the components of RAS, their tissue-specific expression, and how they may change under pathological conditions. In this review, we discuss classical and novel components of RAS, their role in local tissue physiology, and their changes under specific pathological conditions. A better understanding of local tissue RAS expression and regulation will help achieve more tissue- and condition-specific treatments.

## 2. An Overview of RAS

**Figure 1:**
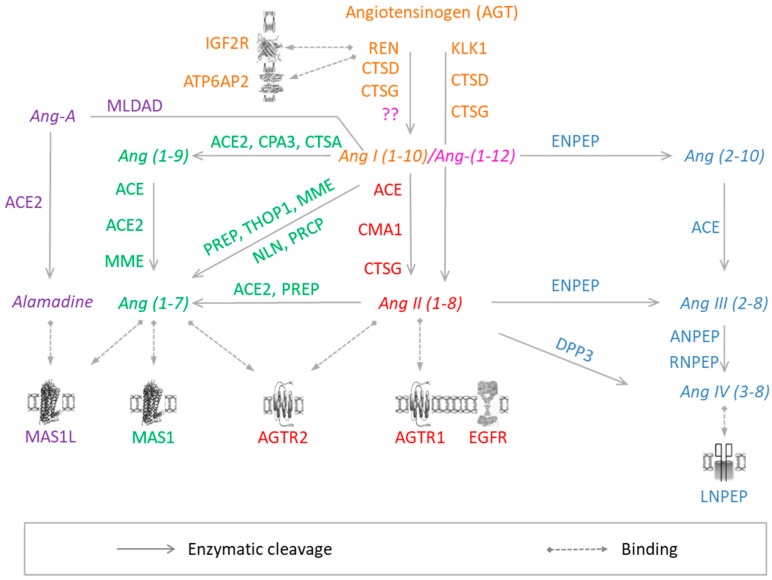
RAS components. Colors correspond to different arms of RAS: Orange, Angiotensin-I; pink, Angiotensin-(1–12); red, Angiotensin-II; green, Angiotensin-(1–7); Blue, Angiotensin III/VI; violet, Alamandine. Proteins are represented by the corresponding official gene symbols. The figure was adapted from Nehme et al. 2015 [7].

In its classical view, RAS includes successive enzymatic reactions resulting in the conversion of the “inactive” substrate angiotensinogen (AGT), into the active peptide angiotensin II (Ang-II) which binds to its specific membrane receptors and elicits cellular effects [2] (Figure 1 and Table 1). AGT is a glycoprotein continuously produced by the liver. In addition, it is differentially expressed in multiple other tissues, including heart, blood vessels, kidneys, and adipose tissue [2]. AGT production can be induced by several stimuli, including inflammation, insulin, estrogen, glucocorticoids, thyroid hormone, and Ang-II [6].

In the plasma, AGT is converted into the decapeptide angiotensin-I (1–10) (Ang-I) by renin (Figure 1 and Table 1), a tightly regulated enzyme produced by the juxtaglomerular cells (JG) [8]. In fact, this step is considered the rate limiting step of Ang-II release in the circulation [8]. Renin is synthesized as an inactive enzyme that is cleaved by microsomes to produce prorenin [9]. Prorenin is then either released as inactive precursor or converted by a variety of proteases into active intracellular renin that is stored in granules of the JG cells. Active renin is released into the circulation by JG cells via an exocytic process and upon a stimulus [8,10] by different mechanisms including Ang-II negative feedback [10].

Ang-I is further processed by angiotensin-converting enzyme (ACE), a membrane-bound exopeptidase, to release the vasoactive octapeptide angiotensin II (1–8) (Ang-II) (Figure 1 and Table 1). Besides Ang-II production, ACE can degrade a number of vasodilating peptides including Ang-(1–7), bradykinin, and kallikrein, thus playing a central role as a vasopressor enzyme [2,8]. Moreover, ACE can activate cellular signaling when bound to its inhibitors (ACEIs) and bradykinin, leading to increased ACE and COX2 production [11].

Ang-II is a biologically active peptide that mediates its effects via the angiotensin-II type 1 receptor (AT1R) [12] (Figure 1). Ang-II was originally known as a circulating hormone that regulates blood pressure and electrolyte balance by acting on vascular contraction, aldosterone secretion, renal sodium handling, sympathetic activity, and vasopressin release [2]. However, molecular studies have shown that AT1R activation can exert long-term genetic effects, in addition to rapid short term effects at the cellular level [13]. Like most other GPCRs, AT1R undergoes rapid desensitization and internalization after agonist stimulation to avoid extensive chronic activation [2].

One of the major effects of Ang-II is the stimulation of aldosterone synthase, CYP11B2, expression in the adrenal cortex [14] (Figure 1 and Table 1). Aldosterone has emerged as an essential regulator of blood pressure in mammals, and has been associated with a variety of diseases in humans [15]. Aldosterone acts in a variety of tissues through its mineralocorticoid receptor (MR) to influence extracellular fluid volume, blood pressure and salt exchange, but may also lead to pathological consequences, mainly tissue fibrosis and oxidative stress [16].

## 3. The Concept of Tissue RAS

Several lines of evidence support the concept of extended RAS that includes multiple synonymous enzymatic pathways for the generation of different angiotensin peptides which exert their effects in a tissue- and condition-specific manner [17]. These pathways may explain the dual role of RAS as not only a circulating hormone, but also a tissue-specific regulatory system serving autocrine, paracrine, and even intracrine functions.

The first demonstration of the presence of a local tissue RAS was in 1971, where a renin-like activity was found in the brain of dogs and which was independent of renin found in the kidney and plasma [18]. This finding was then supported by the identification of Ang-I-like peptides in dog brain with variable molecular weights [19]. Since then, local angiotensin pathways and their physiological importance were elucidated in different tissues including the heart, blood vessels, kidney, brain, adipose tissue, adrenal gland, pancreas, liver, reproductive system, lymphatic tissue, placenta and the eye (Table 2) [2,20,21]. In these tissues, local RAS acts independently from systemic RAS in a paracrine and autocrine manner, but may still interact with systemic RAS to exert endocrine effects [2]. A study conducted by Lau et al. showed a local angiotensin-generating system in the exocrine pancreas. Their data showed the existence of an islet angiotensin-generating system that has an important role in physiological regulation of glucose-induced insulin secretion [22]. In addition, recent studies have reported the expression of renin and angiotensinogen genes and identified their products at many local tissue sites, which further supports the concept of multiple tissues synthesizing RAS components [23]. In fact, multiple studies have described tissue RAS and reported its role in various tissues such as cardiac, vascular and renal tissues, which have the majority of ACE in the body. It seems that tissue RAS has long-term effects on cardiovascular function and structure, while its alteration can cause pathologic conditions [24].

### 3.1. Angiotensin-I

Apart from Renin, several enzymes were found to cleave angiotensinogen into Ang-I, such as cathepsin D (CTSD), cathepsin G (CTSG), and tonins [25] (Figure 1). In addition, studies have shown that three main receptors can be bound and activated by renin, which are: renin-binding protein (RnBP), mannose 6-phosphate/insulin-like growth factor II (M6P/IGFII) receptor and the renin/prorenin receptor (R/PR) [26] (Figure 1 and Table 1). Although RnBP is known to be a renin inhibitor, the latter two are known to increase renin catalytic activity and activate intracellular signaling [20]. R/PR binding is supposed to induce full non-proteolytic activation of prorenin by a conformational change, through which prorenin active site is exposed [26]. The interaction between R/PR and prorenin is species-specific, which may explain the lack of rat prorenin activation by human R/PR [27].

The concept of local RAS was initially challenged by the fact that renin was considered the rate-limiting specific enzyme to cleave AGT into Ang-I [28]. However, renin mRNA and/or activity was detected in several extra-renal tissues, including the vascular wall [29], cardiac [30], adipose [31], and eye tissues [32]. In addition, M6P/IGFII receptor, encoded by the IGF2R gene, contribute to the uptake and activation of M6P-containing prorenin from the circulation at different tissues and in a variety of cells including cardiomyocytes [33], fibroblasts, VSMCs, and ECs [26,33]. On the other hand, the P/PR was shown to be expressed and active in diverse tissues, including the kidney, vascular wall, brain, and the eye. Alterations in R/PR are associated with pathologies such as glomerulosclerosis, diabetes, hypertension, neovascularization and inflammation [26].

In fact, renin is considered the rate limiting enzyme for the generation of Ang-I in plasma, whereas in tissues, enzymes other than renin are thought to regulate Ang-I generation [34]. Of importance is CTSD, which is a ubiquitous lysosomal aspartyl protease [35] that was shown to provide an alternative angiotensin production pathway after myocardial infarction, and hence falsely increase clinical plasma renin activity determinations [36]. Cathepsin D knockdown by siRNA led to more than a two-fold reduction in intracellular and extracellular Ang-I and Ang-II production by VSMCs under both normal and high glucose concentrations [37]. Interestingly, however, silencing of tissue plasminogen activator (tPA) gene, previously associated with Ang-II production [38], moderately increased intracellular Ang-I and Ang-II levels in VSMCs only under high glucose concentrations, while strongly decreased Ang-I and Ang-II levels in the media [37]. However, a consensus on the renin-compensatory activity of CTSD has not been reached yet due to the low specificity and efficiency of CTSD, which has been found to only metabolize AGT under acidic conditions [39,40].

Despite the importance of this rate limiting step in RAS, little effort has been made to further support local Ang-I generation from renin or renin-like enzymes. Therefore, further studies should be performed to identify local mechanisms involved in angiotensinogen cleavage, either through renin recruitment from the circulation, or through local renin-independent enzymatic reactions.

### 3.2. Angiotensin (1–12)

One of the recent important discoveries in RAS is the identification of the Angiotensin (1–12) peptide (Ang-(1–12)), which may serve as an alternative precursor for the production of bioactive angiotensin peptides, independent from Ang-I production [41]. Ang-(1–12) was first isolated by Nagata et al. from rat small intestine, and was shown to induce constriction of aortic strips ex vivo and to raise blood pressure in rats when infused intravenously [41]. Both the vasoconstrictor and pressor responses to Ang-(1–12) were abolished by ACE inhibitors (ACEI) and AT1R blockers (ARBs), which suggest a renin-independent pathway for angiotensin peptides production [41,42]. Despite the differences in amino acid sequence of rat and human Ang-(1–12), Ferrario et al. showed cardiac production of Ang-(1–12) in a rat model expressing human AGT [43].

In addition, compelling evidence suggests that Ang-(1–12) is a major source for local Ang-II production in the central nervous system. Endogenous neutralization of Ang-(1–12) using antibodies directed against the C-terminal end of Ang-(1–12) into a lateral cerebral ventricle of (mRen2)27 transgenic hypertensive rats prompted blood pressure reduction that was associated with a transient anti-dipsogenic behavior [44]. Central effects of Ang-(1–12) were later shown to be mediated via the Ang-II/AT1R axis in the solitary tract nucleus and hypothalamic arcuate nucleus [45,46,47]. Ang-(1–12) was also shown to be present abundantly in a wide range of rat organs and tissues, including heart ventricular myocytes, small intestine, spleen, kidneys, and liver [41]. Lower levels were also found in the medial layer of intracoronary arteries and vascular endothelium [48]. Despite the increase in plasma renin activity following low-salt feeding, the levels of Ang-I, Ang-II, and Ang-(1–12) in plasma and various tissues remained unchanged [49], which suggests that Ang-(1–12) metabolism is regulated in a manner that is independent of circulating renin activity.

Studies on Ang-(1–12) clearly demonstrate species-specific, tissue-specific, and condition-specific metabolism that favors one pathway or enzyme, over the others [17]. For instance, Jessup et al. showed that Ang-(1–12) immunoreactivity was detected in both the heart and kidney of spontaneously hypertensive rats (SHR) and Wistar–Kyoto (WKY) rats. However, tissue measurements by radioimmunoassay showed higher cardiac and lower renal levels of Ang-(1–12) in SHR, compared with WKY rats [48]. It was shown that Ang-(1–12) was cleaved by ACE to generate both Ang-I and Ang-II in rat serum, independent of renin participation [50]. On the other hand, the same team showed that Ang-(1–7) and Ang-(1–4) were the main products of Ang-(1–12) metabolism in renal cortical membranes of rats, which were abolished by neprilysin inhibition [50]. In addition, myocytes of WKY rats were shown to sequester Ang-(1–12) in culture, which was mainly metabolized by ACE and membrane metallo-endopeptidase (MME) [51]. Interestingly, the uptake and metabolism were higher in cardiomyocytes obtained from SHR rats with a predominant effect of chymase in these cells [52]. On the other hand, chymase was shown to be the major enzyme contributing to Ang-(1–12) cleavage in an isolated heart model of cardiac ischaemia-reperfusion injury in Sprague–Dawley rats [53]. ACEI, however, but not chymostatin, inhibited circulatory Ang-(1–12) production in both SHR and WKY rats^42^.

Ang-(1–12) production and metabolism have raised concerns about the possible role of this peptide in mitigating the effects of renin and ACE inhibitors in the treatment of heart failure, which warrant further studies to identify tissue-specific RAS metabolic targets for disease treatment.

### 3.3. Angiotensin-II

In addition to the ACE-dependent cleavage of Ang-I, Ang-II can also be produced by the direct cleavage of AGT by cathepsin G [54], tonin, and kallikrein, or through Ang-I cleavage by chymase and cathepsin G^216^ (Figure 1 and Table 1). Of importance, chymase, a serine protease that is highly specific to the Phe^8^-His^9^ bond [17] of Ang-I, was shown to be more active than ACE in generating Ang-II in human heart [55] and diabetic kidneys [56].

In addition to AT1R (Figure 1 and Table 1), Ang-II also acts through AT2R, a seven transmembrane receptor that acts mainly through Gi and tyrosine phosphatases to exert inhibitory actions on cellular responses mediated by AT1R, mainly by inhibiting cell growth and proliferation while promoting cell differentiation, in addition to vasodilation and reducing blood pressure [57].

Ang-II was thought for a long time to be only a circulating peptide, exerting its effects through endocrine mechanisms. However, many studies identified Ang-II in several tissues and showed that it was produced locally independent of systemic RAS. The first demonstration of tissue Ang-II was in the arterial wall in sheep in 1980 [58]. Studies quantifying tissue Ang-II synthesis, using radiolabeled angiotensin, revealed that Ang-II in the heart, kidney, and adrenal gland [59,60] almost completely originates from local synthesis, both under normal and pathological conditions [60,61]. ACE was shown to be expressed in multiple tissues, including vascular endothelium, renal proximal tubular endothelium, heart, lung, small intestine, colon, activated macrophages, and several regions of the brain [62], where physiologic effects of ACE are the result of tissue rather than circulating ACE activity [63].

ACE is generally considered the main Ang-II-forming enzyme in the circulation. However, in tissues, various serine proteases were shown to play a role in Ang-II generation [53]. Not only chymase, but also trypsin [64] and kallikrein [65] serine proteases were shown to generate Ang-II in vitro and in vivo in ischemic dog hearts, ischemic human hearts, and even in normal healthy individuals during exercise [66]. In fact, chymase has been a focus of interest because of its specificity and potency in the human cardiovascular system [55,67].

Ang-II may exert local paracrine or autocrine effects through its locally expressed AT1 and AT2 receptors (Figure 1 and Table 1). AT1R was found to be expressed in several adult tissues, including blood vessels, heart, kidney, adrenal glands, and liver [2]. On the other hand, AT2R is mainly expressed in fetal tissue and decreases through fetal development [2] to be restricted to certain tissues, mainly the heart, vessels, brainstem, liver, and kidney [68]. At the tissue level, AT1R and AT2R exert opposite effects; therefore, the final local effects of Ang-II are defined by the combined net result obtained from the activation of both receptors. For instance, AT1R induces vasoconstriction and sodium retention in the kidney whereas AT2R promotes vasodilation and natriuresis [69]. On the other hand, in the gastrointestinal tract, AT2R opposes the actions of AT1R in sodium and water absorption, which contributes to the regulation of the finely tuned sodium transport in this tissue [70]. In general, AT2R stimulates protein dephosphorylation, which counterbalances protein phosphorylation induced by AT1R, thus, affecting the signaling pathways inside the cell, leading mainly to opposite cellular actions [69]. Despite this general “antagonistic” view of AT2R, certain studies on cardiac myocytes showed that its overexpression may complement, rather than antagonize, the AT1R effects in cellular hypertrophy [71,72]. Similarly, AT1R and AT2R synergistically act to induce adipogenesis and lipid storage in adipose tissue, wherein AT1R inhibit lipolysis, while AT2R induces the expression of lipogenic enzymes [73]. Interestingly, this adds a new level of complexity to RAS in which the effects of the system depend not only on the “inter-molecular” balance between the antagonistic arms, but also on the “intra-molecular” balance of the levels of the same molecule in certain cells under specific conditions.

Ang-II is the most studied pathway in RAS and additional research on it would open new avenues in understanding the complexity of the system and inter-pathway interactions.

### 3.4. Angiotensin-(1–7)

Ang-(1–7) was first discovered in rat brains in 1983 by Tonnaer and his colleagues [74]. However, at that time, it was thought to be an inactive peptide. The importance of Ang-(1–7) emerged in 1988 when it was found to be the major Ang-I-derived peptide in the presence and absence of ACE inhibition [75]. Ang-(1–7) was initially thought to exert its hypotensive effects in a bradykinin-dependent manner [76]. However, it was later demonstrated that Ang-(1–7) opposes the vasoconstrictive and proliferative actions of AT1R-mediated Ang-II actions [2,17]. In fact, the discovery of Ang-(1–7) and its effects lead to the belief that RAS local actions are mainly driven by the balance between the vasoconstrictor/proliferative and vasodilator/anti-proliferative actions of Ang-II and Ang-(1–7), respectivley [2].

Ang-(1–7) can be formed by different enzymes and pathways (Figure 1 and Table 1). The most potent and well known Ang-(1–7)-generating enzyme is ACE2 (angiotensin-I converting enzyme 2), which can generate Ang-(1–7) directly from Ang-II, or indirectly from Ang-I through Ang-(1–9) intermediate [77,78]. In fact, the former pathway is more favorable because the affinity of ACE2 to Ang-II is 400-folds greater than that to Ang-I [79]. Ang-(1–9) can be generated from Ang-I by the action of ACE2, cathepsin A (CTSA) [80], or carboxypeptidase A3 (CPA3), and then cleaved to form Ang-(1–7) by ACE [77], ACE2, or neprilysin (MME) (Figure 1 and Table 1). Alternatively, Ang-(1–7) can also be formed directly from Ang-I by prolylendopeptidase (PREP), thimet Oligopeptidase 1 (THOP1), [81] and Neurolysin (NLN) or from Ang-II cleavage by ACE2, PREP, and prolylcarboxipeptidase (PRCP) [2,79] (Figure 1 and Table 1).

In fact, ACE2 levels and the ACE/ACE2 ratio is generally considered a reference for Ang-(1–7) production. However, ACE2 is restricted to certain tissues and cells such as endothelial cells of the heart, kidneys, and testes [82]. In addition, the contribution of alternative enzymes in the production of Ang-(1–7) should be considered. For instance, metallopeptidase activity accounts for almost all Ang (1–7) production in atrial homogenate preparations, whereas Ang-II was produced equally by ACE and chymase while cathepsin A was responsible for 65% of the liberated Ang (1–9) [80]. This indicates that local angiotensin peptides production depends as well on the activity of “alternative” enzymes at the tissue level.

Ang-(1–7) exerts its effects mainly through the Mas receptor (MasR) (Figure 1 and Table 1). MasR was first described as Ang-(1–7) receptor in 2003, where its deletion abolished the binding of Ang-(1–7) to mouse kidneys, accompanied with the loss of Ang-(1–7)-induced relaxation [83]. By binding to MasR, Ang-(1–7) may induce many effects, antagonizing those of Ang-II/AT1R, such as vasodilation, inhibition of cell growth, anti-thrombosis, and anti-arrhythmogenic effects [84]. In addition, it was shown that MasR may antagonize AT1R in vitro and in vivo by forming a hetero-dimer with the AT1R, thus blocking the latter’s activity [85]. Moreover, Ang-(1–7) can act on the AT2R (Figure 1 and Table 1), which exerts very similar effects to those induced by MasR [86]. In addition, emerging evidences raised controversies on the specificity of MasR to Ang-(1–7). Recently, MasR was shown to be stimulated by multiple other molecules such as Neuropeptide FF, Alamandine, Angiotensin III, Angiotensin IV, and Angioprotectin. Similarly, independent studies demonstrated the absence of MAS1 activation after Ang-(1–7) treatment in human mammary arteries from patients undergoing coronary revascularization surgery, splanchnic vessels from cirrhotic liver of human and rats and aorta from Sprague–Dawley rats [87].

Ang-(1–7) is present in the circulation, in addition to several other tissues and organs including the heart, blood vessels, kidney and liver [88], where it exerts local paracrine and autocrine actions. The alteration in circulatory and tissue Ang-(1–7) levels were shown to be associated with several diseases, including hypertension preeclampsia, hypertrophic myocardial disease, cognitive heart disease, myocardial infarction (MI), chronic kidney disease (CKD), and hepatic cirrhosis [2]. For instance, *ACE2^−/−^* mice developed age-dependent cardiomyopathy with increased oxidative stress, neutrophilic infiltration, inflammatory cytokine, and collagenase levels, mitogen-activated protein kinase (MAPK) activation and pathological hypertrophy [89]. These effects were inhibited by irbesartan, an AT1R blocker (ARB), which indicates a critical role for ACE2 in the suppression of Ang-II-mediated heart failure. In addition, a recent study suggested an important role for the ACE/ACE2 imbalance in the pathogenesis of severe acute pancreatitis where the ratio of pancreatic ACE2 to ACE expression was significantly reduced and paralleled the severity of the disease [90]. In another study, a reduction in ACE/ACE2 ratio was shown to be associated with acute respiratory distress syndrome, which was prevented by Ang-(1–7) or ARB treatment [91]. Recent studies have supported a metabolic role for the Ang-(1–7)/MasR arm in the liver and its counter-regulatory action to Ang-II/AT1R that interferes in several steps of intracellular insulin signaling arm in the pathophysiology of liver diseases [92]. Indeed, Ang-(1–7) has been shown to ameliorate glucose tolerance and to enhance insulin sensitivity, while Mas receptor has been described as an essential component of the insulin receptor signaling pathway [93]. Of interest, ACE2 treatment has been shown to ameliorate liver fibrosis through reduction of hepatic Ang-II levels concomitant with increased concentrations of Ang-(1–7) in liver tissue [94,95,96]. Moreover, Ang-(1–7)/MasR axis agonists may also play a role in the treatment of CKD by controlling the inflammatory response and fibrosis in kidney tissue [97].

Of note, high concentrations of Ang-(1–7) exerts biphasic effects on Na^+^-, K^+^-ATPase activity in a dose dependent manner by inducing similar effects to those induced by Ang-II at high concentrations, independent of MasR and AT2R, probably through the AT1R [98]. However, in the presence of Ang-II, Ang-(1–7) antagonized the stimulatory effects of Ang-II on Na^+^, K^+^-ATPase activity through a A779-sensitive receptor [99]. On the other hand, Ang-(1–7) infusion or MasR deficiency enhanced renal damage in models of renal insufficiency by aggravating the inflammatory response through NF-κB [100]. In contrast, another study showed that Ang-(1–7) suppressed inflammation by inhibiting the NF-κB pathway in rats with permanent cerebral ischaemia [101].

Taken together, these studies suggest that Ang-(1–7) exerts cell-specific effects based on its concentrations, available receptors, angiotensin peptides, and the physiological state of the tissue.

### 3.5. Angiotensin-III/-IV

Arterial concentration of Ang-III (hexapeptide 2–8) was first documented in 1980 in sheep, and accounted for 42% of that of Ang-II [58]. Ang-III is generated from Ang-II by the removal of the first amino terminus aa by Aminopeptidase A (ENPEP) [8] (Figure 1 and Table 1). In addition, it can be generated from Ang-I by a two-steps pathway involving ENPEP and ACE, respectively [102]. Studies have shown that Ang-III exerts similar, but less potent, actions as compared to Ang-II [2,103], by acting on AT1R and AT2R, with higher affinity to the former [104]. Indeed, Ang-III was shown to increase blood pressure, vasopressin and aldosterone release, in addition to inducing inflammatory genes expression [2,103].

Ang-III in turn can be converted into Ang-IV (pentapeptide 3–8) by the action of the aminopeptidase N (ANPEP) [105], and possibly aminopeptidase B (RNPEP) [106] (Figure 1 and Table 1). Ang-IV acts through its Angiotensin type 4 receptor (AT4R), which is the insulin-regulated membrane aminopeptidase (IRAP). The latter is a type II integral membrane spanning protein belonging to the M1 family of aminopeptidases that is expressed in several tissues, including the brain, adrenal gland, kidney, lung, liver, and heart [107] (Figure 1 and Table 1).

Recent studies have shown that certain local Ang-II-mediated effects could be attributed to Ang-III. For example, Padia et al. showed that the conversion of Ang-II to Ang-III is critical for AT2R-mediated natriuresis in Sprague–Dawley rats [104]. Similarly, in Wistar rats, the Ang-II-mediated enhancement in baroreceptor heart reflex was abrogated in the presence of ENPEP inhibitor, indicating that Ang-III is the active angiotensin peptide involved in central blood pressure regulation [108]. On the other hand, Handa et al. showed that intrarenal injection of Ang I, Ang-II, or Ang-III induce dose-dependent vasoconstriction in Sprague–Dawley rats. However, Ang-IV or Ang-(3–10) injection produced a dose-dependent rapid vasoconstriction, lasting for seconds, followed by a transient vasodilatation, lasting for minutes [109]. This indicates that RAS induces peptide-specific effects at the tissue level.

The major effects of AT4R activation are thought to be in the brain where it can enhance learning and memory [107]. However, the mechanism by which Ang-IV exerts its effects through IRAP is still not clear [110]. One suggestion is that Ang-IV inhibits the catalytic activity of IRAP, thereby extending the half-life of its neuropeptide substrates. Another suggestion is that it may modulate glucose uptake by modulating GLUT4 trafficking. Others suggest that it may act directly on cellular mechanisms by inducing cellular signaling after its binding [110]. Ang-IV in the brain was also shown to be implicated in regulating blood pressure by acting on the AT1R [111], which was shown to mediate several Ang-IV effects. Indeed, Ang-IV mediates pressure and renal vasoconstrictor effects in mice via AT1a receptor whereas AT4R is not involved [112]. Finally, Ang-IV-mediated non-prostaglandin renal vasodilatory activity was found to be linked to renal vascular AT1R [109].

The Ang-III/Ang-IV axes have added a new level of complexity to the system and identified novel mechanisms by which Ang-II may exert its effects. This needs to be further studied to elucidate possible flows in the interpretation of the effects of Ang-II agonists and to identify possible mechanisms that would improve Ang-II antagonists’ mode of action.

### 3.6. Angiotensin A/Alamandine/MrgD

Ang-A is a recently discovered angiotensin peptide detected in the plasma of patients with end-stage renal disease, where Ang-A/Ang-II ratio was found to be higher compared to healthy individuals [113]. Ang-A is an octapeptide with the sequence Ala-Arg-Val-Tyr-Ile-His-Pro-Phe, which can be produced from Ang-II by conversion of the first amino acid, aspartic acid, into alanine [113]. Ang-A can bind to AT1R and AT2R with equal affinity as Ang-II [114]. Intravenous and intrarenal administration of Ang A induced dose-dependent increase in blood pressure and renal vasoconstrictor responses in normotensive and spontaneously hypertensive rats [114,115]. In isolated perfused rat kidney, Ang-A induced smaller vasoconstrictive effects compared to Ang-II, which were inhibited using AT1R inhibitor, but not AT2R inhibitor [113,114].

In fact, the importance of Ang-A is increasing due to its junctional position in the system. Despite its vasoconstrictive and pro-proliferative actions, Ang-A is also a precursor of alamandine, a recently discovered peptide identified in rats, mice, and humans [116]. Alamandine can also be produced from Ang-(1–7) by ACE2 and was shown to produce several physiological actions that resemble those produced by Ang-(1–7) including vasodilation, antifibrotic, antihypertensive, and CNS effects, independent of MasR and AT2R [116]. The effects of alamandine were shown to be mediated through the activation of a specific receptor, the member D of Mas1-related G-protein-coupled receptor (MasDR) [116], which has been recently demonstrated to also be an alternative receptor to Ang-(1–7) [117]. Interestingly, alamandine was shown to exert opposite effects in central nervous system where microinjection of alamandine into the rostral ventrolateral medulla of rats induced a vasopressor effect, whereas its administration into the caudal ventrolateral medulla elicited a vasodilatory effect. Of importance, similar effects were obtained after Ang-(1–7) injection [116]. On the other hand, in control but not diseased blood vessels, alamandine enhanced acetylcholine-mediated vasodilation in normal thoracic aorta and the iliac artery, whereas it reduced it in the renal artery [118]. Interestingly, these effects were absent in blood vessels from atherogenic rabbits, which also showed a reduced vasoconstrictive response toward Ang-A.

The finding of MasDR receptor has added another level of complexity into RAS, especially with regards to the anti-inflammatory Ang-(1–7) axis. This warrants further studies that may explain additional interactions and would balance between the different axes of RAS.

### 3.7. Other Angiotensin Peptides

Ang-(1–9) was considered for a long time as an intermediate peptide with no biological significance. However, recent evidence suggests that Ang-(1–9) can exert several effects in vivo and in vitro independent of Ang-(1–7)-mediated MasR activation, possibly through AT2R [2]. Indeed, a new study showed that Ang-(1–9) exerts beneficial cardiovascular effects via the AT2R in hypertensive rats independent of blood pressure modulation, where it ameliorated structural alterations (hypertrophy and fibrosis) and oxidative stress in the heart and aorta and improved cardiac and endothelial function [2]. These effects were inhibited by an AT2R antagonist, but not a MasR one. On the contrary, in another study on rats, Ang-(1–9) enhanced thrombosis, decreased plasma concentrations of tissue plasminogen activator (tPA), and increased the levels of its inhibitor (PAI-1) through indirect activation of AT1R [119]. These effects were reversed by selective antagonists to AT1R, but not to that of Ang-(1–7).

Ang-(3–7) is an angiotensin peptide that was shown to bind to AT4R, with lower affinity compared to Ang-IV, leading to important effects in the brain and kidney [2]. Ang-(3–7) can be produced by cleavage of Ang-(1–7), Ang-II, or Ang-IV by aminopeptidases or carboxypeptidases [2]. Administration of Ang (3–7) intracerebroventricularly (i. c. v.) significantly enhanced learning and behavioral activity in rats [120]. Co-treatment withthe ARB, losartan, only affected learning ability, without altering the behavioral activity. This suggests that Ang (3–7) is an active peptide that exerts its effects through different receptors, one of which is AT1R [120]. Moreover, Ang-(1–7) induced inhibitory effects on the energy-dependent solute transport in proximal tubules of the rat kidney [121] were shown to be mediated by the metabolism of Ang-(1–7) into Ang (3–7), by binding to AT4R [121]. Such results may raise questions about the previously described “direct” effects of certain angiotensin peptides.

## 4. Conclusion and Future Directions

**Figure 2:**
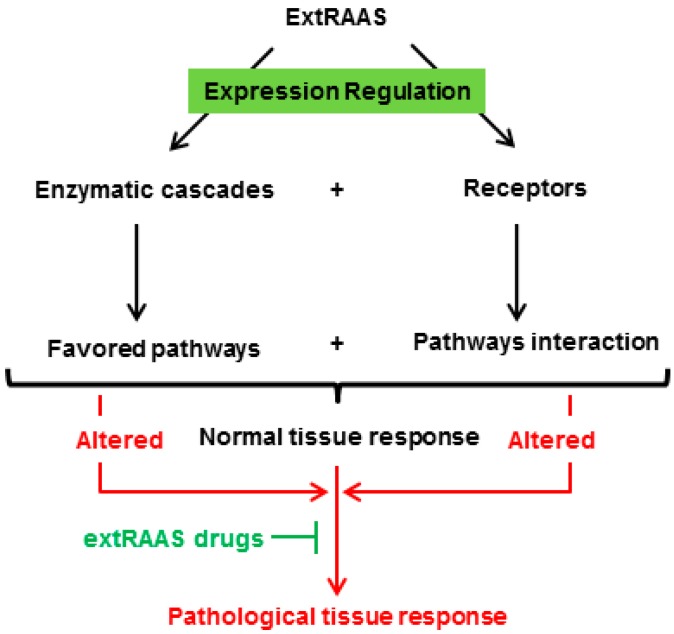
A specific combination of locally expressed RAS enzymes in a tissue results in the production of a specific combination of peptides that can bind to their corresponding receptors, leading to a locally balanced paracrine/autocrine effect that plays a role in tissue physiology and homeostasis. A change in local balance of RAS components will consequently lead to pathophysiological consequences.

The concept of tissue RAS could be defined as a specific combination of RAS enzymes that are locally expressed in a tissue, which results in the production of a specific quantitative and qualitative combination of peptides that can bind to their corresponding locally expressed receptors, thus leading to a locally balanced paracrine/autocrine effect that plays a role in tissue physiology and homeostasis. A change in local RAS expression will consequently lead to alterations in the balance obtained, and thus, to pathophysiological consequences (Figure 2). In this regard, studies on RAS need to be shifted from the one peptide-one pathway approach, toward a more general approach that considers the tissue-specific pathways and their respective local and systemic interactions. Indeed, the knowledge obtained from the former approach may lead to misleading conclusions that rely on the used model, with a lack of information on other pathways that may balance the effect of the pathway in question. Therefore, for a better understanding of the “real” global physiological effects of RAS, it is necessary to measure the different components of RAS in a specific tissue, under specific physiological conditions.

Using transcriptomics meta-analysis, we have recently established the atlas of tissue RAS, which includes the transcriptional maps of RAS in 23 normal human tissues [7,122]. The maps provide information on the favored pathways of RAS in each tissue, but also on the co-expression of RAS genes, which may provide the basis for the discovery of potential regulatory mechanisms involved in the global expression of RAS components at the tissue level. In this regard, we have recently created the transcriptional maps of RAS in normal and atherosclerotic vascular wall showing the differences in angiotensin metabolism between both tissues [123]. Also, by analyzing the promoters of co-expressed genes, we identified potential transcription factors that could play a role in the global expression of RAS components in atheroma. Therefore, the atlas needs to be extended and studied at the protein level. In addition, RAS maps should be established from studies on each tissue under pathophysiological conditions, which will help understand the way the system is altered in each tissue under specific conditions, and thus, a better understanding of the mechanisms by which the system is involved in local tissue pathophysiology.

## Figures and Tables

**Table 1:** Extended renin-angiotensin-aldosterone system components.

Gene Symbol	Gene Description	Gene ID
ACE *	angiotensin I converting enzyme (peptidyl-dipeptidase A) 1	1636
ACE2	angiotensin I converting enzyme (peptidyl-dipeptidase A) 2	59272
AGT *	angiotensinogen (serpin peptidase inhibitor, clade A, member 8)	183
AGTR1 *	angiotensin II receptor, type 1	185
AGTR2	angiotensin II receptor, type 2	186
ANPEP	alanyl (membrane) aminopeptidase	290
ATP6AP2	ATPase, H+ transporting, lysosomal accessory protein 2	10159
CMA1	chymase 1, mast cell	1215
CPA3	carboxypeptidase A3 (mast cell)	1359
CTSA	cathepsin A	5476
CTSD	cathepsin D	1509
CTSG	cathepsin G	1511
DPP3	dipeptidyl-peptidase 3	10072
EGFR	epidermal growth factor receptor	1956
ENPEP	glutamyl aminopeptidase (aminopeptidase A)	2028
IGF2R	insulin-like growth factor 2 receptor	3482
KLK1	kallikrein 1	3816
LNPEP	leucyl/cystinyl aminopeptidase	4012
MAS1	MAS1 oncogene	4142
MME	membrane metallo-endopeptidase	4311
NLN	neurolysin (metallopeptidase M3 family)	57486
PREP	prolyl endopeptidase	5550
REN *	renin	5972
RNPEP	arginyl aminopeptidase (aminopeptidase B)	6051
THOP1	thimet oligopeptidase 1	7064
AKR1C4	aldo-keto reductase family 1, member C4	1109
AKR1D1	aldo-keto reductase family 1, member D1	6718
CYP11A1	cytochrome P450, family 11, subfamily A, polypeptide 1	1583
CYP11B1	cytochrome P450, family 11, subfamily B, polypeptide 1	1584
CYP11B2 *	cytochrome P450, family 11, subfamily B, polypeptide 2	1585
CYP17A1	cytochrome P450, family 17, subfamily A, polypeptide 1	1586
CYP21A2	cytochrome P450, family 21, subfamily A, polypeptide 2	1589
GPER	G protein-coupled estrogen receptor 1	2852
HSD11B1	hydroxysteroid (11-beta) dehydrogenase 1	3290
HSD11B2 *	hydroxysteroid (11-beta) dehydrogenase 2	3291
NR3C1	nuclear receptor subfamily 3, group C, member 1 (glucocorticoid receptor)	2908
NR3C2 *	nuclear receptor subfamily 3, group C, member 2 (Mineralocorticoid receptor)	4306

* Classical RAS components.

**Table 2:** The physiological and pathophysiological role of RAS in different tissues.

Tissue	Physiological Role of RAS	Associated Diseases
Blood vessel	Vasomotor regulation, oxidative metabolism	Hypertension, atherosclerosis
Heart	Vasomotor tone, fibrotic regulation, oxidative metabolism	Heart failure, cardiac hypertrophy and fibrosis
Kidney	Blood pressure regulation	Chronic kidney disease
CNS	Sympathetic regulation of blood pressure	Hypertension
Adipose tissue	Adipogenesis	Insulin resistance and obesity
Eye	Aqueous humor dynamics	Glaucoma and diabetic retinopathy
Liver	Glucose metabolism	Glucose intolerance and fibrosis
